# Atomic Level Insight into Wetting and Structure of Ag Droplet on Graphene Coated Copper Substrate—Molecular Dynamics versus Experiment

**DOI:** 10.3390/nano11061465

**Published:** 2021-06-01

**Authors:** Aleksandra Drewienkiewicz, Arkadiusz Żydek, Marcela E. Trybula, Janusz Pstruś

**Affiliations:** 1 Institute of Metallurgy and Materials Science, Polish Academy of Sciences, 30-059 Krakow, Poland; a.drewienkiewicz@imim.pl (A.D.); a.zydek@imim.pl (A.Ż.); j.pstrus@imim.pl (J.P.); 2 Biological and Chemical Research Centre, University of Warsaw, 02-089 Warsaw, Poland

**Keywords:** molecular dynamics, graphene, wetting, copper, silver, Voronoi analysis, contact angle, wettability, sessile drop method

## Abstract

Understanding the atomic-level phenomena occurring upon the wetting of graphene-coated Cu with liquid Ag is pivotal for the description of the wetting phenomenon and the role of graphene as a diffusion barrier. We have performed molecular dynamics (MD) simulations and confronted with our present experimental results to characterize wetting behavior of graphene coated Cu surfaces. Perfect and defected graphene layers covering Cu surface were wetted with liquid Ag droplet at 1273 K. Structural and topological aspects are discussed to characterize structure of the liquid Ag droplet and a product of wetting reaction occurring on Cu/Gn and Cu/Gn_def_ substrates, also including perfect graphene layer and a pure Cu surface. The obtained results reveal the importance of defects in graphene structure, which play a key role in wetting mechanism and the formation of AgCu alloy. As a consequence, we observe a change of the wetting behavior and topology of both bulk and adsorbed Ag atoms by using Voronoi analysis (VA). Despite the differences in time scale, atomistic simulations allowed us to catch the early stages of wetting, which are important for explaining the final stage of wetting delivered from experiment. Our findings reveal also graphene translucency to metal-metal interactions, observed in previous papers.

## 1. Introduction

Graphene is known from its extraordinary properties, which have allowed it to find a broad spectrum of applications ranging from electronics to medicine as well as gain significant research interests (over 38,000 scientific papers on graphene in 2020). Potential applications include sensors, capacitors, energy storage devices, solar cells, transparent conductive coatings, and tissue engineering applications [1,2,3]. Graphene can also serve as a diffusion barrier against chemical compound formation on metallic surfaces, as it was already portrayed in several examples [4,5,6]. Ideal structure of graphene sheet is impermeable even to the smallest atoms, like He or H, and provides long-term protection [7]. Despite the effectiveness of chemical or physical graphene fabrication methods, defects have been found in experimentally produced graphene [8,9,10]. Prasai et al. showed that multiple graphene layers can offer better protection against a corrosive liquid environment than a single graphene layer [11].

Diffusion at the solid/liquid interface plays a huge role in electronic packaging industry, where tin-silver-copper (SAC) solders are mostly used as joints. They form intermetallic compounds (IMCs) with substrates like Cu or Ni. The extensive growth of IMCs is unwanted due to worsening joint properties and enhancing the risk of fracture, which is dangerous for electronic devices [12,13]. As yet, no solution has been given and there is a need to find a way to provide at least partial joint protection from undesirable effects. Graphene can be a potential solution the joint protection problem because it can be used as a diffusion barrier for substrates, on which it was directly deposited or transferred.

In this context, there is a gap in the knowledge about the structure and properties of metal/graphene interfaces as well as wetting of graphene-coated metallic substrates with a metal droplet. So far, few papers exist which provide knowledge about graphene layer and metal/graphene interface wetted with water requiring a further explanation [14,15,16]. Lai et al. tried to clarify the inconsistencies existing in the literature about graphene wetting by arguing that they are result of time dependence of graphene wettability which could not be captured at macroscale [17]. Contrary to this, limited examples for wetting with liquid metals are found [18,19,20]. Ko et al. [19] provided a first experimental insight into a mechanism of the intermetallic phases (IMCs) formation on a defected graphene coating on a Cu substrate. Kinetics of this process occurring on a Cu/Gn interface is slower and leads to suppressed IMCs growth at the Cu/graphene interface. Later studies of Pstruś et al. [20] delivered information about the structure of CVD-grown graphene on a Cu substrate, which consists of “flakes” separated by boundaries being a diffusion path for a solder to migrate towards the Cu substrate. The existing graphene flake boundaries enhance wettability properties of Cu/graphene substrate. Similar observations were also reported in experimental works of Sobczak et al. [21] and Homa et al. [22] who studied an interaction occurring at the Cu-graphene/liquid Sn interface and the SiC-graphene/ liquid Ag interface, respectively. In the first case, the importance of the graphene layer discontinuities was discovered which allow liquid Sn to permeate through a graphene layer and form IMCs with Cu. While the results obtained for liquid Ag droplet unveiled a graphene etching phenomenon, leading to both carbon diffusion during wetting of solid substrate and a local graphene reorganization near the Ag droplet surface. Interestingly, Homa et al. [23] discovered the transport of a double graphene layer on the top surface of droplet after wetting. This phenomenon is a result of mass transfer observed through the graphene-droplet interface. So far, the number of experimental works providing full description of metal-graphene substrate wettability with liquid metal is limited to a few papers, while no atomic level understanding has been given. Therefore, atomistic simulations and DFT-based calculations can be helpful in understanding of the wetting phenomenon and its impact on structure and properties.

Atomic-scale physical models, such as molecular dynamics and Monte Carlo methods, offer a possibility of gaining insight into the wetting phenomenon, interface structure, and kinetics of potential reactions on atomic level. They involve physical and chemical equations for describing interatomic interactions, which are then pivotal for a proper description of the wetting reaction on metal and metal/graphene substrates. Only a few computational papers can be found for graphene-coated substrates as compared to a graphene layer wetted by liquid metal droplets. Caccia et al. performed ab initio calculations for a study of wetting translucency in Au droplet on a pure and graphene-supported 4H-SiC substrate as well as graphene substrate with variable layer number [24]. As a result, graphene was found to be translucent with respect to Au/SiC interactions due to a strong interaction between SiC and Au droplet surfaces, which is partially shielded by graphene layer/s. Importantly for our present work, Kumar et al. recently studied ideal graphene sheet with fixed C atoms, wetted with silver nano droplet at temperatures ranging from 300 K to 1500 K [25]. He used the gold standard Lennard-Jones potential to describe a weak Van der Waals interactions between carbon and silver atoms. The present work is motivated by the absence of atomic level understanding of wetting mechanism, structure and mechanism of silver droplet spreading on graphene-coated Cu substrate.

In this work, we provide the first atomic level insight into a wetting mechanism, structure, and topology of a silver droplet wetting graphene-coated Cu substrate at 1273 K. The aim of our paper is to unveil the mechanism of graphene wetting with liquid Ag droplet, and the structure of both Cu/graphene interface and the liquid droplet. For comparison, pure Cu substrate and a graphene layer are also considered. Here we present molecular dynamics simulation results and experimental observations. To get a more detailed insight into the mechanism of graphene wetting and its better understanding, we monitored changes of contact angle with time and provided information on structural and topological aspects to better understand the behavior of liquid droplet and the wetting phenomenon. 

## 2. Materials and Methods

### 2.1. Molecular Dynamics Simulations (MD)

Molecular dynamics simulations have been performed for the study of structure, wetting properties and spreading mechanism of silver droplet on Cu/graphene substrate at 1273 K. We have considered four different substrate variants wetted with a silver droplet. They are pure Cu, graphene layer (Gn), Cu substrate covered by both a perfect graphene (Cu/Gn) and defect containing graphene layers (Cu/Gn_def_). In the last case, two atomic columns in the center of the graphene layer were removed. Depending on the interaction type, we used charge-optimized many body potential (COMB3) formalism [26] for describing the interaction for Cu/graphene interface, including Cu-Cu, Cu-C and C-C atomic pairs, as implemented in the LAMMPS code [26]. The COMB3 formalism includes all necessary interatomic interactions for a metal-nonmetal system, it has been described in detail in papers [27,28,29]. Importantly, we used the COMB3 potential parameterization recently provided by Klaver et al. [30].to study the Cu/graphene interface. They clearly proved the suitability of COMB3 formalism for Cu/graphene description, which we do not repeat again in this work. The COMB3 [26,31] and Reactive Force field (ReaxFF) formalisms [32,33] are commonly used for describing chemical reactions on metallic substrate [34,35,36,37,38], which in principle account for hybrid interaction types being of critical for a wetting phenomenon on metal-graphene substrate.

For describing metal-metal interaction (Ag-Ag and Cu-Ag), the embedded atom model (EAM) potential was used [39], with parameters provided by Williams et al. [40]. The EAM potential is a gold standard force field used mostly for description of the interaction in metallic materials [41,42]. After a recent paper by Kumar [25], we used the 12-6 Lennard-Jones (*L*-*J*) potential for describing the van der Waals interaction between liquid Ag droplet and graphene layer:

(1) $$E_{LJ}=4\varepsilon_{Ag-C}\left[{\left(\frac{\sigma_{Ag-C}}{r}\right)}^{12}-{\left(\frac{\sigma_{Ag-C}}{r}\right)}^{6}\right]$$

The interaction energy (*ε_Ag-C_*) and interatomic distance (*σ_Ag-C_*) between Ag and C were 0.0301 eV and 3.006 Å, respectively, as standard numerical values reported in the literature [25].

### 2.2. MD Simulation Set-Up

In the first step of our MD simulation procedure, both Cu matrix and graphene (Gn) layer were separately heated from 0 K to 1273 K before wetting studies, and then both were thermalized during 20,000 ps with 0.5 fs time step. Cu matrix with approximately 100 Å × 100 Å × 30 Å dimensions was tightly covered by a graphene layer of similar dimensions in the xy plane on which periodic boundary conditions were imposed. The Cu substrate contained 20,000 atoms, the Gn layer 7500 atoms, the Gn_def_ layer 7364 atoms and the Ag droplet contained 1678 atoms. These numbers were constant for each initial configuration considered. To prepare the Ag droplet, a cubic 30 Å × 30 Å × 30 Å box containing Ag atoms was heated above the Ag melting point (1400 K) until a droplet was formed. In the second step, the Ag droplet was placed about 7 Å over the four different substrate variants. MD simulations with canonical ensemble (NVT) were performed at 1273 K for three substrate variants with maximum simulation time up to 0.5 ns, and 1 ns for Cu/Gn_def_/Ag system. In the case of variant of graphene substrate with Ag droplet, rigid body assumption was made for graphene layer during whole simulation time. Nose-Hoover thermostat, implemented in LAMMPS code, was used for maintaining a target temperature during whole simulation. The visualization of wetting phenomenon occurring for four substrates variants was made by using Visual Molecular Dynamics (VMD) package [43].

Selected atomic structures of each considered substrate variant wetted with Ag droplet were minimized using conjugated gradient method, as implemented in LAMMPS, before performing structural and topological analyses as well as calculating wettability. Time dependence of contact angles (CA) was computed for four configuration variants used to describe wetting properties of Cu matrix covered by a graphene and a single graphene substrate. Spreading area and the height of droplet on a substrate were also determined using MD simulation derived structures and compared with experimental data. Procedure of CA determination is described in the modelling section of the Supporting Information.

Voronoi analysis is based on three dimensional Voronoi tessellation, which provides a detailed description of atom linkage in 3-dimensional space. It gives an deep insight into local atom changes through a complete geometrical construction of atom to its neighbor atoms. More details about VA can be found in papers [44,45,46]. Voronoi index (*n*3, *n*4, *n*5, *n*6, *n*7) is used to differentiate the types of VC, where *ni* is the number of i-edged sides in the polyhedron. This tool allows to recognize body- and face-centered cubic structures (bcc and fcc), as well as hexagonal close-packing (h.c.p).

Wettability was measured by determining spreading area *P_av_*, and its coefficient *K_p_*, according to a formulation proposed by Pstruś et al. [47]. For this calculation, we have considered Ag atoms adsorbed on four different considered substrates. At the first stage, ellipse shape was assumed for determining area for adsorbed Ag atoms on a substrate, and minimum and maximum values of X axis and Y axis were correspondingly chosen for calculation of ellipse area. At the next stage, *P*_0_ was needed, which is a projection of a sphere on the surface, to compute *K_p_* coefficient. We considered atomic structure of Ag droplet in Gn/Ag variant at 1 ps, and then computed the mean value of sphere area by using the formula: $P_{0}=\pi \times r^{2}$. The Equation (2) was used to calculate the *K_p_* coefficient as follows:

(2) $$K_{p}=\left(P_{av}-P_{0}\right)/P_{0}$$

Similar methodology was used to calculate *K_h_* coefficient, where the radius of the sphere was already known (*D*_0_). The height of the droplets on considered variants were calculated based on maximum and minimum value of Z for Ag atoms of geometries at 1200 ps. *K_h_* coefficient was computed using formula:

(3) $$K_{h}=\frac{D_{0}-H_{St}}{D_{0}}*100\%$$

### 2.3. Experiment

A Chemical Vapour Deposition method was used for graphene growth on Cu foil with the dimensions of 30 × 30 × 0.25 mm and its purity of 99.999% (Alfa Aesar, Kandel, Germany). We followed step-by-step a procedure of graphene deposition as it is described by Pstruś et al. [20]. Ag sample of 0.3 g and 99.99% purity (INNOVATOR Sp. z o.o., Gliwice, Poland) was put onto Cu/graphene substrate and then placed in a device used for sessile drop method SD at cold zone, which was constructed at IMMS PAS. The entire system was closed and purged with argon atmosphere. The furnace was turned on and the samples were transported to the heating zone after reaching temperature of 1273 K. Since Ag sample had melted, time was measured during 30 s and then the sample was moved to the cold zone. More details about the experimental procedure is described in the Supporting Information. Wettability tests were performed for a Cu/Gn/Ag sample and the spreading area was computed using ImageJ software [48] for top view of the sample image after wetting experiment. The procedure of calculation was repeated 10 times in order to produce reliable results, which were then used for calculating the *K_p_* coefficient.

## 3. Results and Discussion

### 3.1. Wetting Mechanism on Cu/Gn and Cu/Gn_def_ Substrates

**Figure 1:**
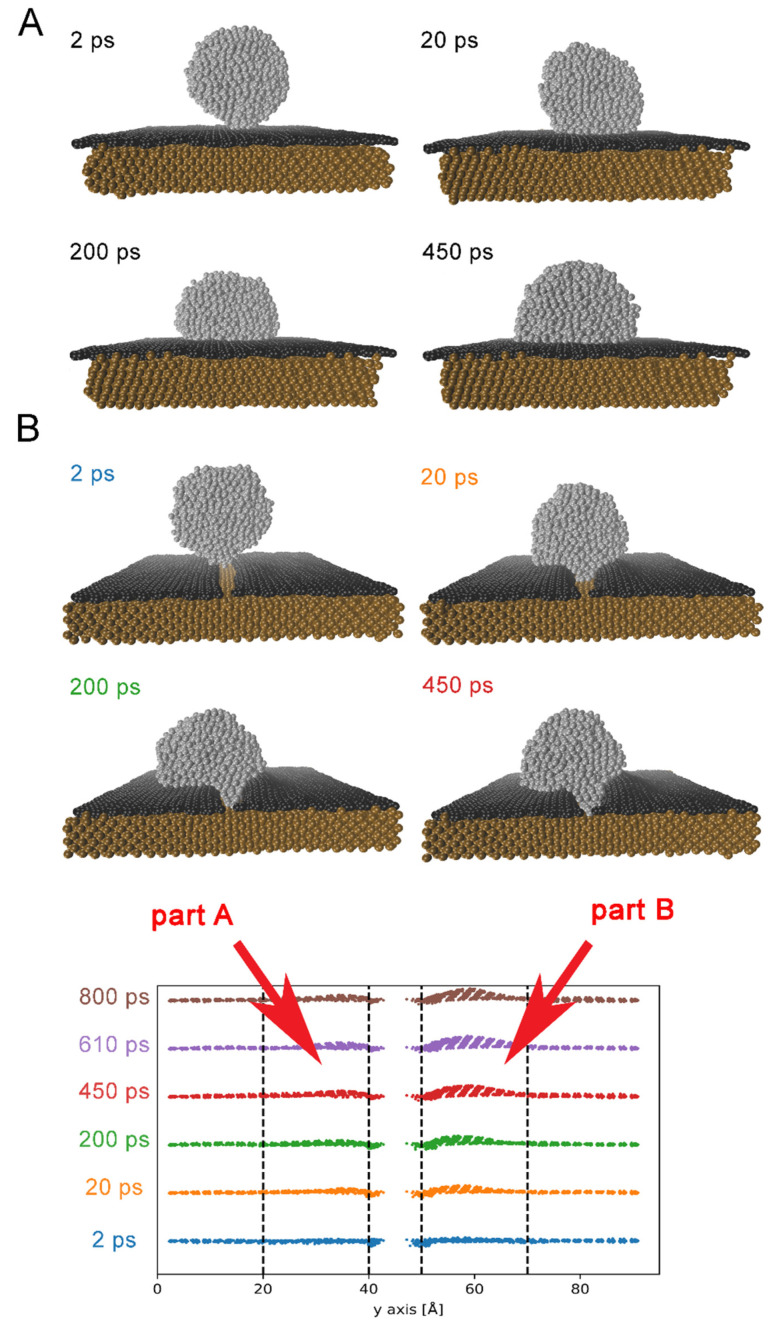
Snapshots taken from MD simulations present wetting phenomenon occurred on (**A**) Cu/Gn substrate and (**B**) Cu/Gn_def_ substrates at 1273 K. Silver, black and orange color represent Ag, graphene and Cu atoms, respectively. Bottom panel presents a time-dependent change of distance between two parts of graphene layer on Cu substrate.

Molecular dynamics simulations results obtained at 1273 K for Ag droplet wetting graphene-coated Cu substrates are discussed below. For clarity of the presentation, we present MD results for Ag droplet wetting a graphene layer and a pure Cu substrate in Figure S1A,B of the Supporting Information, respectively. The evolution of silver droplet spreading on Cu substrate covered by perfect graphene (Cu/Gn) and a defected graphene layer (Cu/Gn_def_) is presented in Figure 1A,B, respectively. Snapshots taken from MD simulations present inward silver atoms diffusion to Cu substrate through defect and Ag droplet starts to spread on two substrates because of surface diffusion, in the course of MD simulations. However, different scenario of wetting mechanism is observed between Cu/Gn and Cu/Gn_def_ substrates.

In case of perfect graphene-coated Cu substrate, silver droplet slowly spreads on Cu/Gn surface and its shape also slowly changes, which is associated with simultaneous diffusion of silver atoms on the top surface of Ag drop. Such behavior illustrates a non-reactive wetting for which a stronger interaction between droplet atoms dominate over the weaker ones between Ag and C. Similar spreading mechanism occurs for Ag wetting a perfect graphene layer as presented in Figure S1B of the Supporting Information. Finally, a hemisphere-like droplet forms on Cu/Gn at 450 ps. For non-reactive wetting, the spreading rate of liquid droplet on substrate is controlled by the viscous flow [49]. On the other hand, non-reactive wetting mechanism requires a precursor foot presence to which belong atoms of liquid droplet extended beyond the circular construction of droplet on a substrate [50] which we do not see it for Cu/Gn/Ag system.

**Figure 2:**
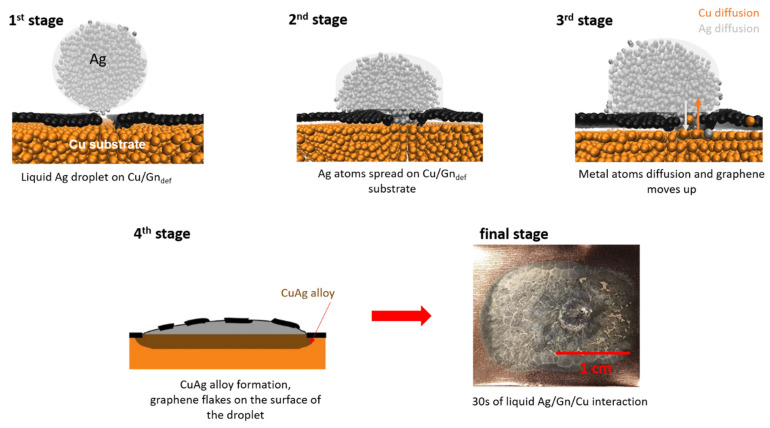
Mechanism of Cu/Gn_def_ substrate wetted with liquid Ag droplet.

Spreading mechanism of Cu/Gn_def_ substrate wetted with liquid silver differs from the simple case described above. A mixture of non-reactive wetting and a reactive wetting is observed. To get deeper insight into these phenomena, we present in Figure 2 a graphical model of Cu/Gn_def_ wetting with liquid Ag droplet by combining atomistic simulations with experiment. At 1st stage, liquid Ag droplet is approaching Cu/Gn_def_ substrate and Ag atoms are getting to interact with Cu atoms through the defect. Simultaneously, an impairment of graphene layers between part A and part B, and surface diffusion of Ag atoms on graphene layer occur at 2nd stage. The bottom part of Figure 1 illustrates the increase of distance between two parts of the graphene layer, and the increasing space between the Cu substrate and the graphene layer close to the defect. The latter can be associated with a feature corresponding to a wrinkle-like formation on a pure Cu substrate, which was previously evidenced through atomistic simulations performed by Klaver et al. [30]. This feature in our present case might result from a weakening of interaction between Cu and C because of a strong interaction between Ag and Cu atoms and inward diffusion of Ag atoms through defect onto the substrate (3rd stage). Ag atoms enter the Cu/Gn_def_ interface and then move on Cu substrate before its dissolution. Consequently, the Cu-graphene distance increases and graphene layer (a flake observed by Pstruś et al. [20]) climbs the liquid Ag droplet surface with simultaneous slow volume diffusion leading to the formation of AgCu alloy at the Cu-graphene interface(4th stage). Final stage of the two simultaneous processes occurring on Cu/Gn_def_ substrate results in a formation of AgCu alloy, as presented in photo taken after 30 s at T = 1273 K. Detailed explanations of graphene “flake” transfer on the top surface of Ag droplet require further studies which are beyond the scope of present paper and will be provided in our next paper. We can see by visual perception silver drop wetted a Cu/Gn substrate and numerous cracks appear on the top surface of Ag drop at final stage (Figure 2). The place of Ag droplet wetting a Cu/Gn substrate corresponds to its initial place and then it can be assumed that the wetting reaction took place in a specific way, during which the Ag droplet did not interact with Cu/Gn substrate for a long time, and suddenly started to interact with Cu substrate because of defect presence. 

Due to the dissolution of carbon in silver, based on the phase diagram for Ag-C [51] and defects accompanied graphene layer, we are supposed to observe the transfer of small parts of graphene layer on the top surface of Ag droplet due to graphene layer imperfection. This process requires longer simulation times, that would explain no change in the position of spreading Ag droplet after completed wetting. After passing a critical point, at which no graphene layer is present, the Ag droplet starts to react rapidly with Cu substrate.

### 3.2. Chemistry of Cu/Gn/Ag and Cu/Gn_def_/Ag Systems

#### 3.2.1. Pair Distribution Function

**Figure 3:**
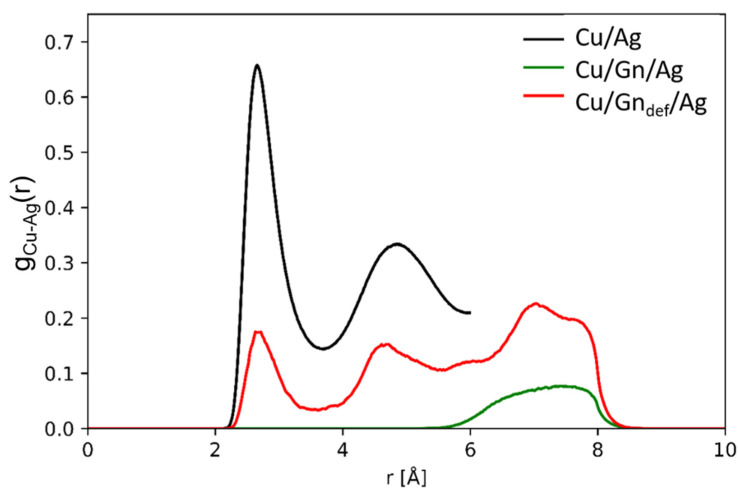
Radial distribution function for Cu-Ag atomic pair computed for Cu/Gn/Ag (green line) and Cu/Gn_def_/Ag (red line) at 500 ps. Results for Cu/Ag systems are added for comparison with an AgCu alloy formed upon reactive wetting of a pure Cu substrate by the liquid Ag droplet.

For a deeper insight into the impact of wetting on the structure of Cu/Gn/Ag and Cu/Gn_def_/Ag systems, we computed radial distribution functions (g_ij_(r)) for each respective atomic pair contributing to the considered substrate variants with Ag droplet. The pair distribution function of the Ag-Cu pair is presented in Figure 3 for MD snapshots taken at 450 ps. For the remaining atomic pairs, Ag-Ag, Ag-C, C-C Cu-C, and Cu-Cu, g_ij_(r) are illustrated in Figure S2A–E in the Supporting Information. For the Ag-Cu pair in the Cu/Gn/Ag system, only one peak between 6 Å and 8 Å appears which is a result of a long-range interaction. In contrast, the g_Ag-Cu_(r) for Cu/Gn_def_/Ag system exhibits three peaks of relatively low intensity as compared to that computed for Ag-Cu pair of AgCu alloy formed upon reactive wetting of a pure Cu substrate (black line, Figure 3). g_Cu-C_(r) for the latter systems was rescaled by 0.1 to be able to make a reliable comparison because of high intensity of the first pronounced peak of g_Cu-C_(r) for Cu/Ag system. Two features require more explanation, namely the positions of the first and third peak of g_Ag-Cu_(r). The first peak of g_Ag-Cu_(r) allocates around 2.9 Å similarly to the position of the first pronounced peak of g_Ag-Cu_(r) for the AgCu alloy (black line, Figure 3). Its position corresponds to Ag-Cu bond length in bulk AgCu alloy [52] while its presence is a significance of reactive wetting occurrence leading to the formation of an AgCu alloy at a place of graphene layer lack for Cu/Gn_def_/Ag system. The third peak is located around 7 Å, which corresponds to a through-graphene interfacial interaction between Ag and Cu observed similarly for Ag-Cu pair in the Cu/Gn/Ag system. Despite alloy formation in the Cu/Gn_def_/Ag system, the bulk Cu substrate remains crystalline, as seen from g_Cu-Cu_(r) in Figure S2E of the Supporting Information. For bare Cu surface, complete reactive wetting leads to the formation of an alloy which is associated with *crystalline-to-amorphous* transformation of the Cu substrate as showed snapshot of spreading evolution for Cu/Ag systems (see Figure S1B) and g_Cu-Cu_(r) drawn in Figure 3 for Cu/Ag system (black line).

#### 3.2.2. Cu-C Bond Distance

**Figure 4:**
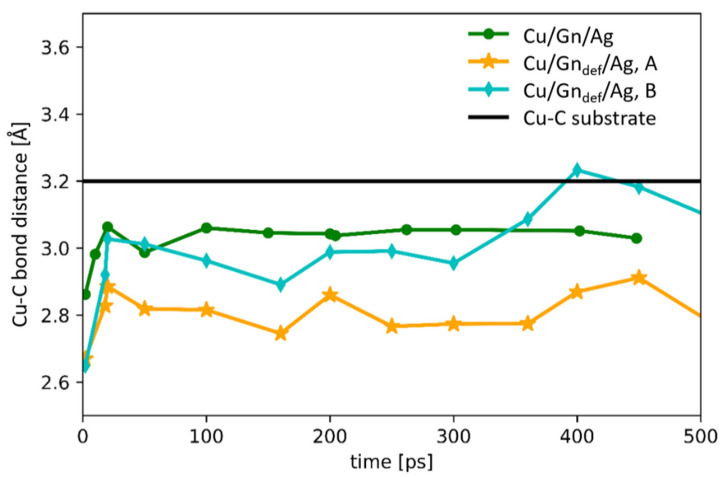
Time evolution of Cu-C bond length Cu/Gn/Ag and Cu/Gn_def_/Ag systems. Green line represents the results for Cu/Gn/Ag system, while part A and part B of Cu/Gn_def_/Ag are illustrated by orange and light blue lines, respectively. Black line was added for a comparison and denotes a reference Cu-C bond distance of Cu/graphene interface.

For a better understanding of graphene layer importance for wetting behavior and the Cu-Ag interfacial interactions in the Cu/Gn/Ag and Cu/Gn_def_/Ag systems, we have also inspected time evolution of Cu-C bond length. Due to the gap dividing the “defected” graphene layer (Gn_def_) into two parts (part A and part B, Figure 1B), we analyzed the Cu-C bond distance for each Gn_def_ part separately. The average values of Cu-C bond length, computed as a minimal distance between graphene layer and outermost Cu containing layer, are drawn in Figure 4 as a function of time.

A logarithmic change of Cu-C bond distance is observed for two Cu/Gn containing substrate variants. For the Cu/Gn/Ag system Cu-C bond distance increases up to 3.04 Å value at which attains a plateau. For the defected graphene layer on Cu substrate, time dependence of the Cu-C bond length is getting more complicated due to a defect presence in graphene and impairment of part A and part B (see Figure 1). Depending on part type of graphene layer, the plateau is observed for different Cu-C bond distances. The Cu-C bond fluctuates around 2.8 Å for part A of defected graphene, while for the second part it is around 3 Å. Such different behavior of Cu-C distance change between two parts of Cu/Gn_def_/Ag system results from an asymmetric shape of Ag droplet formed on the Cu/Gn_def_ substrate. At initial wetting stage, Ag atoms are symmetrically distributed over Cu/Gn_def_ substrate after that Ag atoms start to slowly migrate from part B to part A of Cu/Gn_def_/Ag system and graphene layer of part B is getting wrinkled on the Cu substrate. The Cu-C bond distances in part B are shorter about 0.3 Å from those in part A at 450 ps. As yet, no similar discoveries were provided in the literature, which could support our present findings. Only the experimental study by Homa et al. [23] mentioned above provides a pictorial description of what could happen with a graphene layer at a longer time. Importantly, the Cu-C bond length for the Cu/graphene interface is 3.22 Å according to the recent work by Klaver et al. [30] and our present investigation, which is added to Figure 4 as a reference value. Independently on the graphene layer structure on substrate, the Cu-C bond distance is smaller for two considered graphene-coated Cu substrates covered with liquid Ag droplet on the top instead of its absence. We can observe that the interactions between silver and Cu substrate are getting partially transmitted through the graphene layer, which is visible for the two parts of the Cu/Gn_def_/Ag system as well as for the Cu/Gn/Ag one.

### 3.3. Contact Angle (CA)

**Figure 5:**
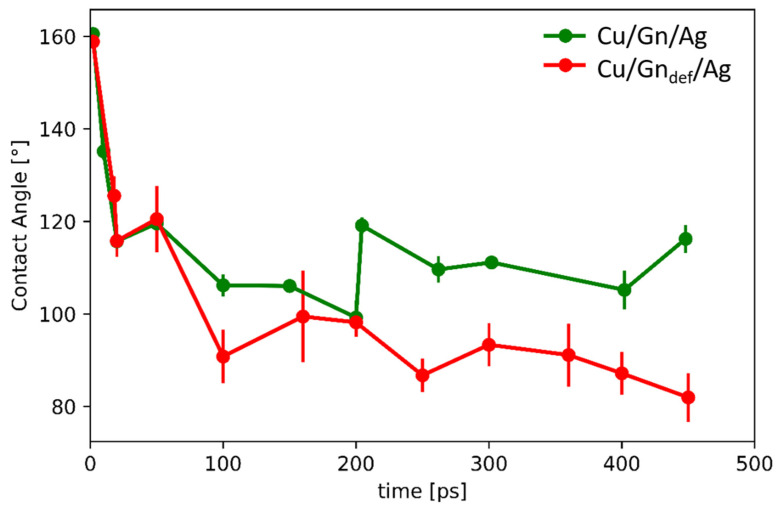
Time evolution of contact angle for Cu/Gn/Ag (green line) and Cu/Gn_def_/Ag systems (red line).

To precisely describe wetting behavior, contact angle was determined from MD simulation results using a procedure described in the the Supporting Information. Time evolution of the contact angle was determined for structures taken at selected simulation times and presented in Figure 5. For both presented systems, the contact angle change depends on graphene layer structure. CA values decrease for the two considered substrates with increasing simulation time, which is more pronounced for the defect containing graphene on Cu substrate. CA of Cu/Gn/Ag decreases attaining plateau around 110°, which for the Cu/Gn_def_/Ag locates around 90° angle at 450 ps. Depending on graphene layer structure, the contact angles obtained for a defected graphene-coated Cu substrate are lower than those for perfect graphene covering a Cu substrate. This trend of CA change is an indicative of an improvement of wettability and deterioration of the diffusion barrier against a strong chemical interaction between Ag and Cu caused by a structural defect incorporated into the graphene layer. Importantly, the surface chemistry of the carbon containing substrate has an impact on the wettability of carbon based materials [53]. A consideration of defected graphene layer covering Cu substrate provides a more realistic picture of wetting behavior which we observe experimentally (see final stage, Figure 2). Due to the difference in time scale, we were not able to capture on atomic scale the moment of graphene layer transfer to the top surface of the Ag droplet. This process requires longer simulation time which is associated with a movement of Ag atoms from part B to part A of Cu/Gn_def_/Ag to be then able to enter Cu/Gn_def_ interface and form an AgCu alloy.

### 3.4. Wettability

#### Spreading of Ag Droplet

**Figure 6:**
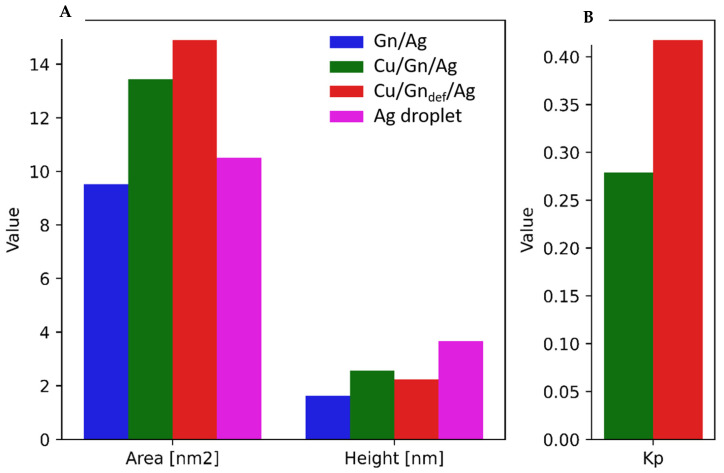
Wettability test for Cu/Gn and Cu/Gn_def_ substrates at 450 ps. (**A**) The spreading area and the height of Ag droplet on two substrates (**B**) *K_p_* spreading coefficient determined for Cu/Gn/Ag and Cu/Gn_def_ substrates and for performed in this work experiment. For a comparison only of spreading area, the results for perfect graphene layer and Ag droplet are also added to part A of figure.

In our further consideration, we focus on performing the wettability test for Cu/Gn/Ag and Cu/Gn_def_ substrates by calculating the size of a droplet area formed upon its spreading on a substrate and the height of droplet after completed wetting. The spreading area for Cu/Gn/Ag and Cu/Gn_def_ substrates and the height of Ag droplet are pictured in the left and the right sides of Figure 6A, respectively. The highest spreading areas are observed for Ag droplet wetting both Cu/Gn and Cu/Gn_def_ substrates as compared to an isolated graphene layer (blue bar, Figure 6A). Spreading area and the height of Ag droplet is given for a comparison in Figure 6A. Such relationship can be an indicative of the graphene translucency to the interfacial interactions between Ag and Cu, which are absent for the isolated graphene layer covered with Ag droplet. In case of defected graphene layer coating Cu substrate, the high value of spreading area for Cu/Gn_def_/Ag system results from the presence of a structural defect in graphene layer and migration of Ag atoms from part B to part A of Cu/Gn_def_/Ag system, which also influences contact angles, as mentioned above. The changes observed for spreading area correlate with those observed for the height of Ag droplet on considered graphene-coated Cu substrates, namely highest spreading area involves a decrease in the height of droplet. While for Gn/Ag system, we found different relationship (see Figure S3B in the Supporting Information) which can then suggest about a liquid phase transformation of Ag droplet due to a presence of contractive interaction between Ag atoms.

Having computed spreading area, we also calculated spreading coefficient, *K_p_*, which is presented in Figure 6B for two considered Cu/graphene based substrates. The spreading coefficient uses spreading area (Figure 6A) and the area of V volume’s sphere projected on a flat surface (see Equation (2)). *K_p_* coefficient increases with the increasing spreading area, and it is higher for defected graphene (red bar, Figure 6B) than for perfect graphene (green bar, Figure 6B) on Cu substrates.

### 3.5. Topological Analysis

#### 3.5.1. Ag Drop Adsorption on Cu/Gn/Ag and Cu/Gn_def_/Ag Systems

**Figure 7:**
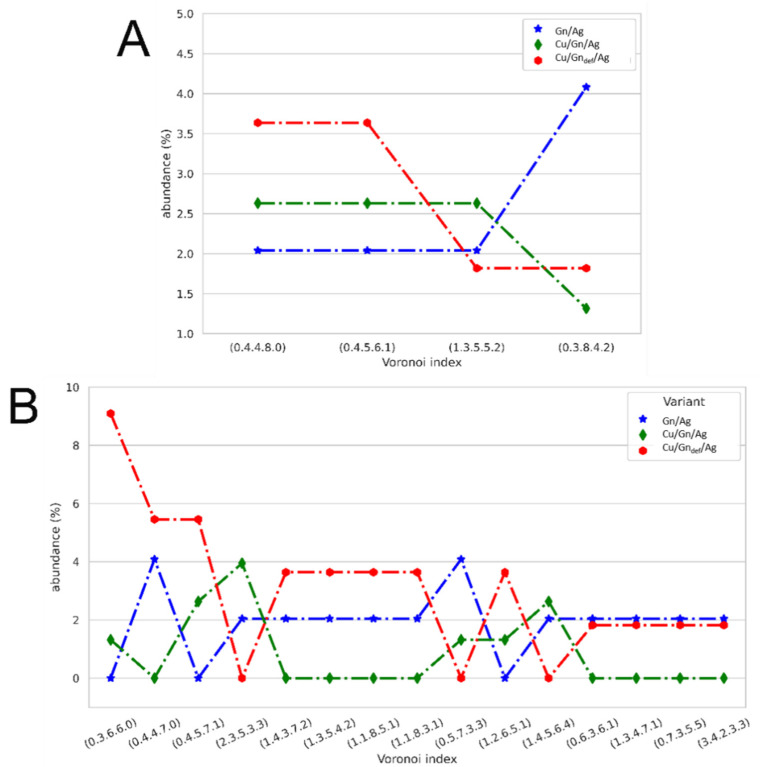
A graph abundance of VC types in considered substrate variants: Gn, Cu/Gn and Cu/Gn_def_. (**A**) VC types found in three substrate variants. (**B**) VC types occurred in two variant.

Looking deeper into the wetting phenomenon, we performed a Voronoi analysis (VA) to characterize the topology of Ag atoms adsorbed on two considered graphene-coated Cu substrates and of Ag atoms in the bulk liquid phase. Abundance graphs of Voronoi cell (VC) types for Ag atoms being in contact with the graphene layer is presented in Figure 7A,B. Depending on the substrate variant, the total number of VC indices found for Ag atoms adsorbed on graphene layer varies between 80 and 150. Depending on their abundance they were divided into three groups. The first group of VC types composes of VC’s present in all three considered substrate variants (Gn, Cu/Gn and Cu/Gn_def_), whose abundance is drawn in Figure 7A The second and third groups consist of VC types simultaneously found in two substrate variants (Figure 7B) and only in one variant (Figure S4A in the Supporting Information), respectively. VA is sensitive to even slightly disturbed atom positions, as we showed in our previous studies [44,45,46]. Topologies of Ag atoms differ between the three groups selected. Importantly, only four VC types of 3, 4 and 5 digit indices are present in all three substrate variants (Figure 7A), as compared to numerous VC types occurring in two and one substrate variant. First group of VC topologies in Figure 7A exhibits about 2.5% abundance of adsorbed Ag atoms on Cu/Gn substrate with (0.4.4.8.0), (0.4.5.6.1) and (1.3.5.5.2) VC topologies, which is a close similar to those Ag atoms on Gn layer. On the contrary, the second group of VC types demonstrates quite different relationship between Ag atoms on Cu/Gn substrate and on Gn layer. The (0.4.4.7.0) 3 digit VC index, which can be associated with f.c.c. lattice distortions in aluminum is of highest abundance in both for Gn layer and Cu/Gn_def_ substrate. Moreover, 5 digit VC indices are also common for both Gn and Cu/Gn_def_, which are of similar abundance to VC indices of 1st group. When analyzing VC types present in one substrate variant presented in Figure S5, we found only VC types occurring either for Gn/Ag or for Cu/Gn_def_/Ag. There is no VC types which would occur only for the Cu/Gn substrate. An abundance graph of VC types detected for Ag atoms forming an AgCu alloy in place of the structural defect in the graphene layer deposited on Cu substrate is presented in Figure S4B of the Supporting Information. It also contains, only for comparison, the abundance of VC types in AgCu alloy formed upon reactive wetting. The VC types found for Ag atoms reacting with Cu are mostly those previously identified for liquid alloys [54,55]. This confirms the occurrence of reactive wetting of C/Gn_def_ substrate leading to alloy formation.

In a view of the performed Voronoi analysis for Ag atoms adsorbed on a graphene layer, we can observe a similarity in behavior of Ag atoms adsorbed on Cu/Gn_def_ substrate to those wetting Gn layer, which could not have been caught by visual perception and even from chemical analysis (Figure 2 and Figure S2A–E of the Supporting Information). This relationship is a result of mentioned above outward movement of the graphene layer from the substrate which appears at the edge of the defect (gap) in the graphene layer.

#### 3.5.2. Topology of Ag Atoms in Droplet after Spreading on Cu/Gn and Cu/Gn_def_ Substrates

**Figure 8:**
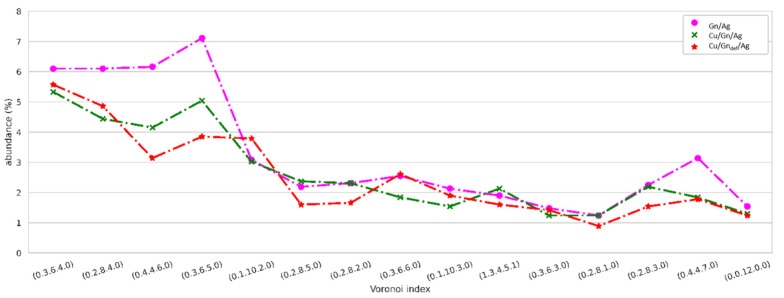
Abundance of 15 most representative VC topologies in liquid Ag droplet on Cu/Gn and Cu/Gn_def_ substrates at 450 ps.

Abundance of 15 most representative VC topologies for bulk liquid Ag droplets wetting Cu/Gn and Cu/Gn_def_ substrates at 450 ps is drawn in Figure 8, while a comparison graph of VC types abundance is presented in Figure S5 in the Supporting Information. The Ag droplets are liquid according to our recent works concerning VC types found in liquid Al- Cu-based alloys [54,55,56], for example. (0.3.6.4.0), (0.2.8.4.0), (0.4.4.6.0) and (0.1.10.2.0) VC indices are of highest abundance as compared to 10 remaining VC types of abundance reaching about 2%. On contrary, they are of low abundance in the AgCu alloy formed upon reactive wetting of the Cu substrate in contact with liquid Ag droplet. There no evidence of unreacted Ag atoms on Cu substrate. Importantly, (0.1.10.2.0), (0.1.10.3.0) and (0.0.12.0.0) VC indices are an indicative of icosahedra presence in liquid Ag droplets, which have recently been a subject of very extensive research performed for amorphous and liquid alloys.

## 4. Conclusions

We have performed molecular dynamics simulations to unveil the mechanism of liquid Ag wetting Cu substrate covered with perfect graphene layer and defect containing one at 1273 K, which is supported with our present experimental result. We have also discussed structural and topological aspects to correctly describe the impact of wetting phenomenon, occurring on different types of substrates. The results clearly show, that the presence of defect in graphene structure affects wettability behavior, and is a pivotal for the occurrence of diffusion as well. Moreover, introducing graphene at the Cu/Ag interface, allows to observe graphene translucency to metal-metal interactions.

Our work shows, that with molecular dynamics we were able to simulate the early stages of wetting of Cu/Gn structure with a silver droplet, observed experimentally. It was achieved mainly due to implementing a defect in graphene in the simulations. Combination of those two approaches allows us to propose a mechanism of wetting, as well as graphene detachment from the substrate, resulting in slowing down the diffusion process in the system. However, several aspects still remain to be considered for a complete description of the wetting process, such as longer simulation time parallel with shortening of the wetting time in the experimental studies. They also concern a consideration of more complex graphene models to MD simulations, including the experimentally-observed defects for CVD-grown graphene layers. Nevertheless, despite the rather simple simulation setup, we were able to obtain consistent results. 

## Data Availability

The data presented in this study are available on request from the corresponding author.

## Supplementary Materials

Supporting Information is available online at https://www.mdpi.com/article/10.3390/nano11061465/s1, Figure S1: Snapshots are taken from MD simulations for Ag droplet spreading on: **SA** graphene layer and **SB** a pure Cu substrate. **SC** Top view of experimentally obtained AgCu alloy droplet, which forms upon reactive wetting of pure Cu substrate. Figure S2 Radial distribution functions of 2**A**: Ag-A; 2**B** Ag-C; 2**C** C-C; 2**D** C-Cu and 2**E** Cu-Cu atomic pairs. Figure S3A Comparison of spreading area between three different substrate variants. Figure S3B Spreading coefficient corresponds to the height of Ag droplet after wetting completed. Figure S4A. A graph abundance of VC types present in one variant for three considered substrate variants Gn, Cu/Gn and Cu/Gn_def_. Figure S4B. A graph abundance of VC types for Ag atoms adsorbed Cu/Gn_def_ and formed an AgCu alloy after reactive wetting is over. Figure S5 Comparison of VC types abundance between three substrate variants and liquid Ag droplet.
