# Supplementary material for: Atomic Level Insight into Wetting and Structure of Ag Droplet on Graphene Coated Copper Substrate—Molecular Dynamics versus Experiment
Source: Nanomaterials (Basel). 2021 Jun 1;11(6):1465. doi: 10.3390/nano11061465 (PMC8228875; doi:10.3390/nano11061465)
Supplement: Supplementary file 1 [file nanomaterials-11-01465-s001.zip › nanomaterials-1225388-supplementary.pdf]

Supporting Information:

Atomic level insight into wetting and structure of Ag droplet on graphene coated copper substrate - molecular dynamics vs experiment

Aleksandra Drewienkiewicz [1], Arkadiusz Żydek [1], Marcela Trybula [1,2,*], and Janusz Pstruś [1]

j1 Institute of Metallurgy and Materials Science, Polish Academy of Sciences, 30-059 Krakow, Poland
2. Biological and Chemical Research Centre, University of Warsaw, 02-089 Warsaw, Poland
* Correspondence: m.trybula@imim.pl

1. Methods

Modelling

Contact angle

The procedure of Contact Angle determination was performed for structures taken from MD simulations at selected simulation time. Contact Angle plug-in was used as implemented in ImageJ Software [1]. First of all, two points at the contact zone between droplet and substrate was chosen creating a baseline. Then, three points along the edge of the droplet was chosen and the "Manual Points Procedure" was used. Ellipse approximation was considered due to lack of axisymmetric droplets. Each calculation was repeated 5 times, and the mean value was calculated from 10 values (each measurement gave left and right CA).

Experimental procedure

Bulk Ag metal with 99,999% purity, produced by Alfa Aesar, were put in a graphite crucible to prepare Ag droplet. Then, it was melted at T = 1273 K in a glove-box with a protective atmosphere of argon (99,9999%), portioned for the use as droplets, and left for 2 hours. Graphene was produced by using Chemical Vapour Deposition (CVD) method. Cu foil, with the dimensions of 30 x 30 x 0.25 mm and the purity of 99,95%, were the substrates for the synthesis. Pads were first rinsed in acetone and then washed with isopropyl alcohol to clean a surface. Then, a procedure of graphene deposition, described in [2], was strictly followed. Next, bare copper foils, as well as those covered with graphene, were used for wetting tests. Onto those substrates, previously prepared Ag droplet was put and the system was heated up to 1000 °C in a furnace, with the use of argon as a protective atmosphere. When the melting process was observed, a time was set to 1 minute. After that time, samples were cooled in a room temperature.

2. Results
   <u>*Wetting of graphene layer and pure Cu substrate*</u>

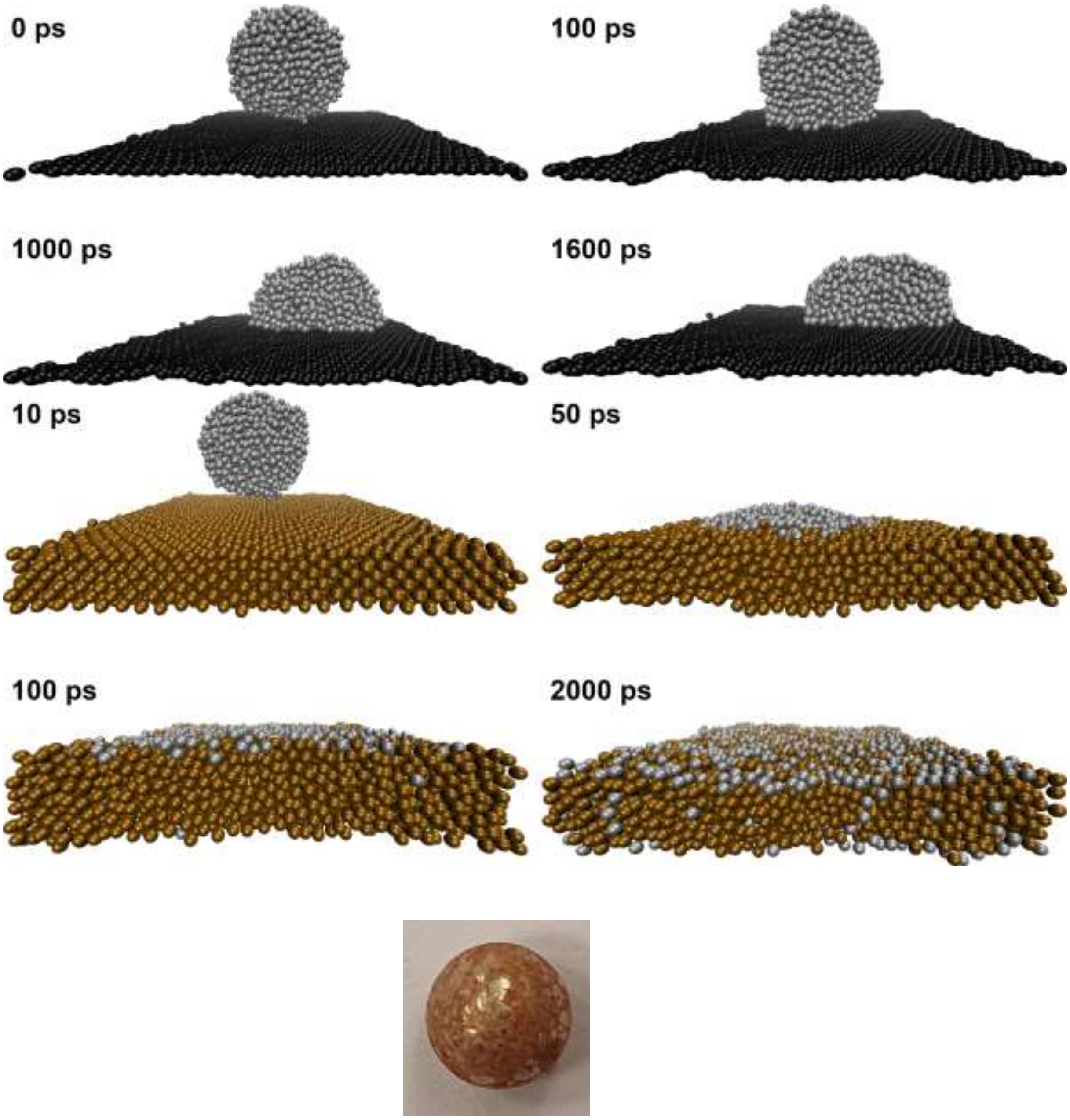

Figure S1. Snapshots are taken from MD simulations for Ag droplet spreading on **A** graphene layer and **B** a pure Cu substrate. C Top view of experimentally obtained AgCu alloy droplet, which forms upon reactive wetting of pure Cu substrate.

Radial distribution functions

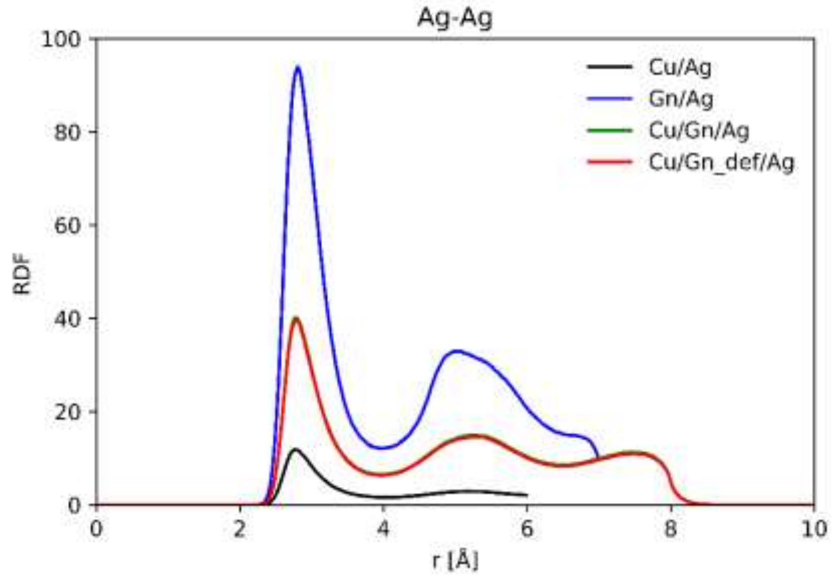

Figure S2A Radial distribution function of Ag-Ag atomic pair computed for Cu/Ag, Gn/Ag, Cu/Gn/Ag and Cu/Gn_{def}/Ag systems formed after spreading of Ag droplet on four respective substrate types. Color of lines corresponds to different substrate variant.

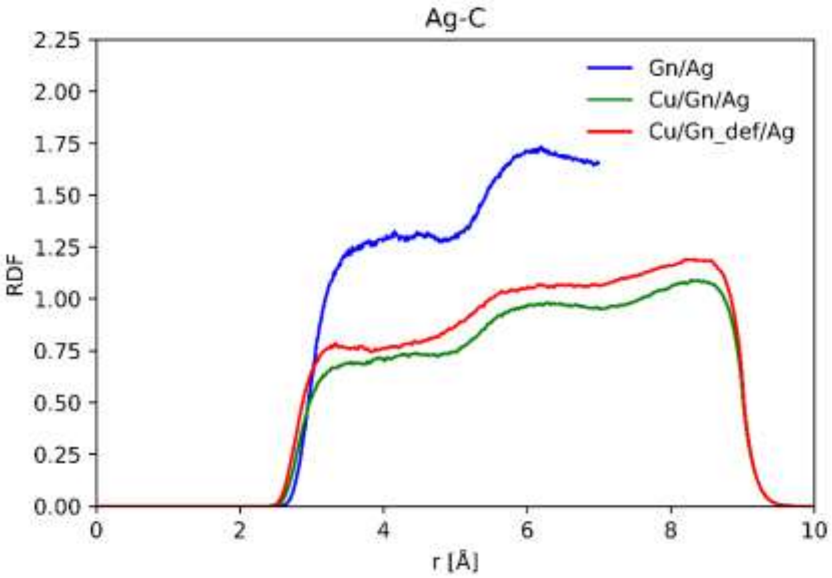

Figure S2B. Radial distribution function of Ag-C atomic pair computed for Cu/Ag, Cu/Gn/Ag and Cu/Gn_{def}/Ag systems formed after spreading of Ag droplet on three respective substrate types. Color of lines corresponds to different substrate variant.

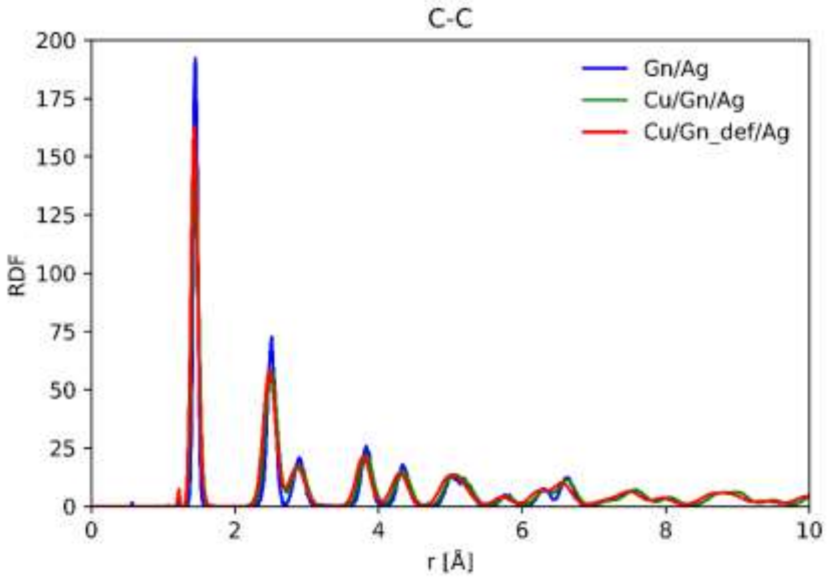

Figure S2C Radial distribution function of C-C atomic pair computed for Gn/Ag, Cu/Gn/Ag and Cu/Gn_{def}/Ag systems. Color of lines corresponds to different substrate variant.

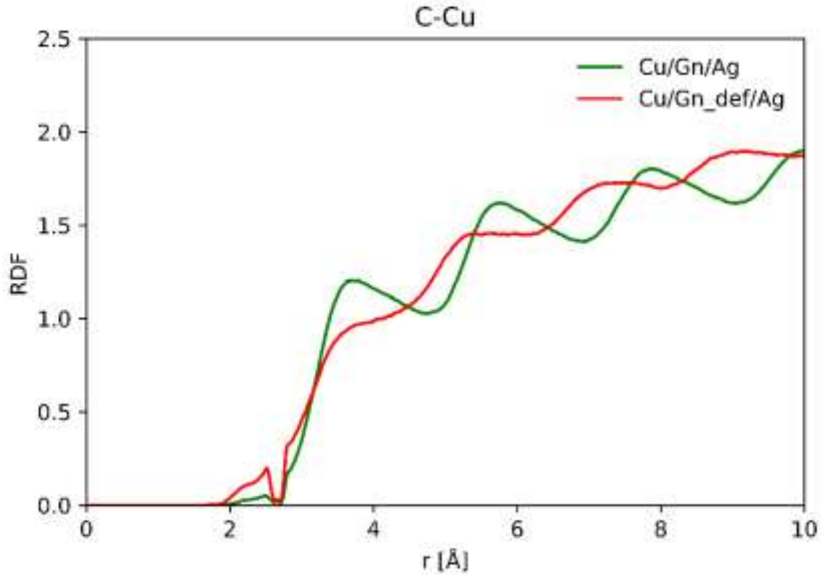

Figure S2D Radial distribution function of C-Cu atomic pair computed for Cu/Gn/Ag and Cu/Gn_{def}/Ag systems. Color of lines corresponds to different substrate variant.

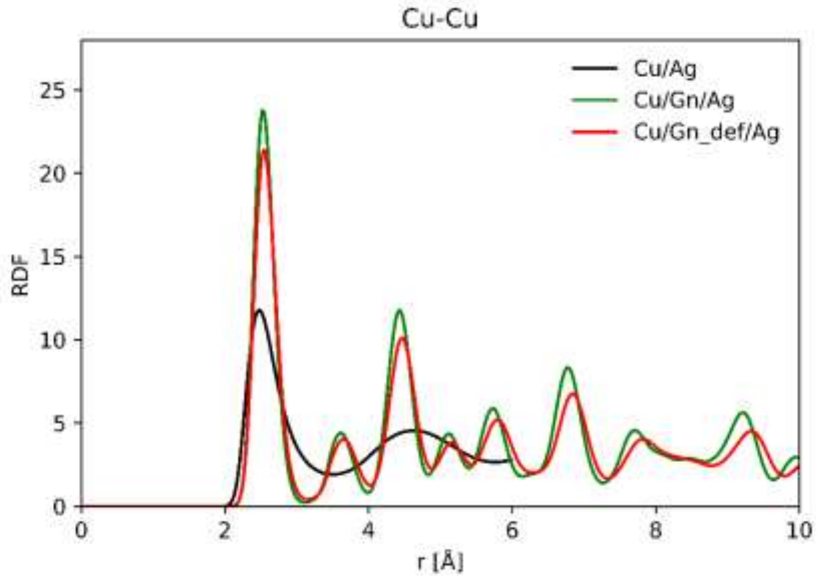

Figure S2E. Radial distribution function of C-Cu atomic pair computed for Cu/Gn/Ag and $Cu/Gn_{def}/Ag$ systems. Color of lines corresponds to different substrate variant.

Spreading area and spreading coefficients

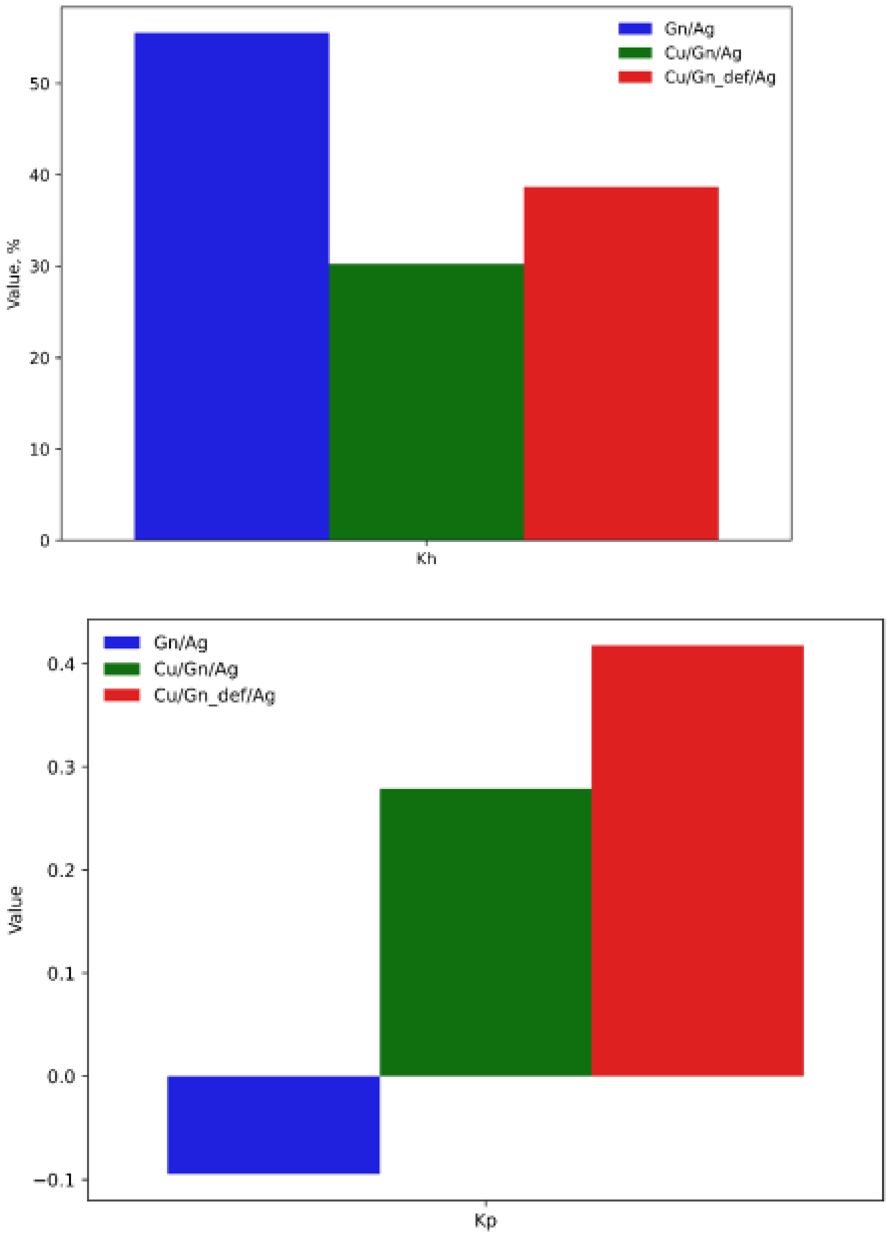

Figure 3 A Comparison of spreading area between three different substrate variants. **B**. Spreading coefficient corresponds to the height of Ag droplet after wetting completed.

Topological analysis of Ag atoms adsorbed on Cu/graphene substrates

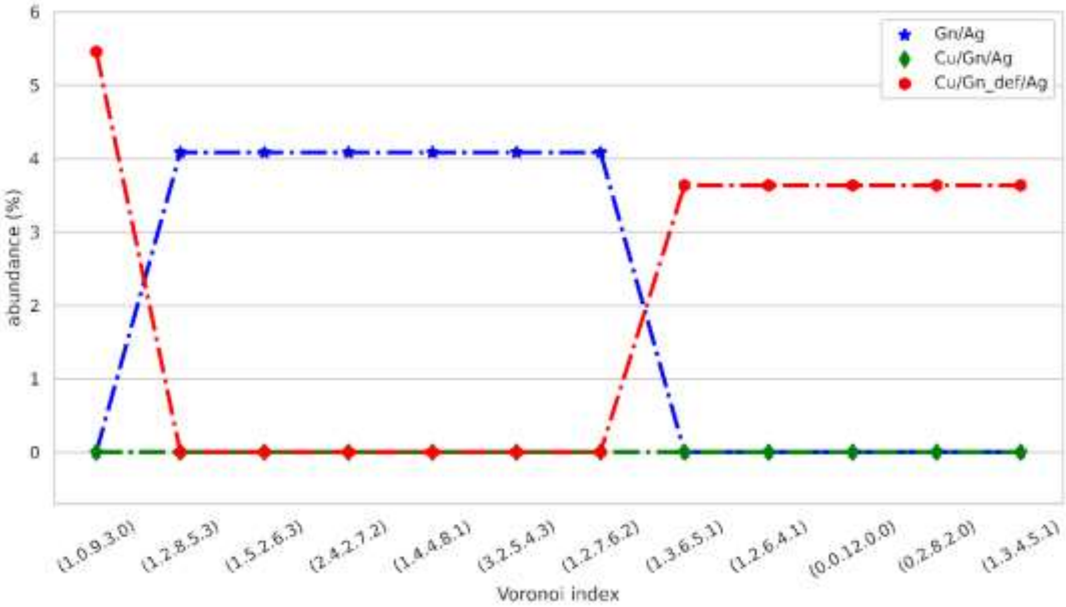

Figure S4A. A graph abundance of VC types present in one variant for three considered substrate variants Gn, Cu/Gn and Cu/Gn_{def}.

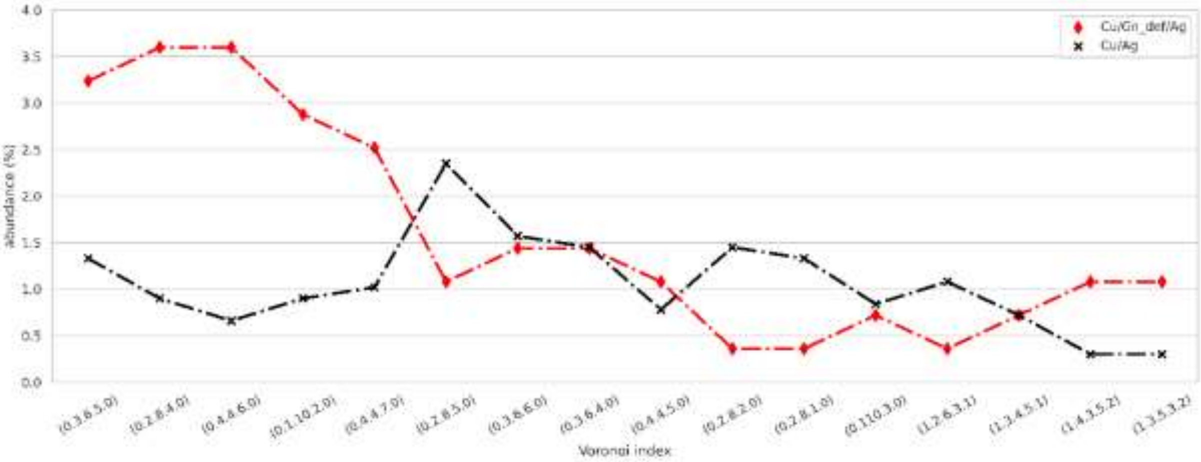

Figure S4B. A graph abundance of VC types for Ag atoms adsorbed Cu/Gn_{def} and formed an AgCu alloy after reactive wetting is over.

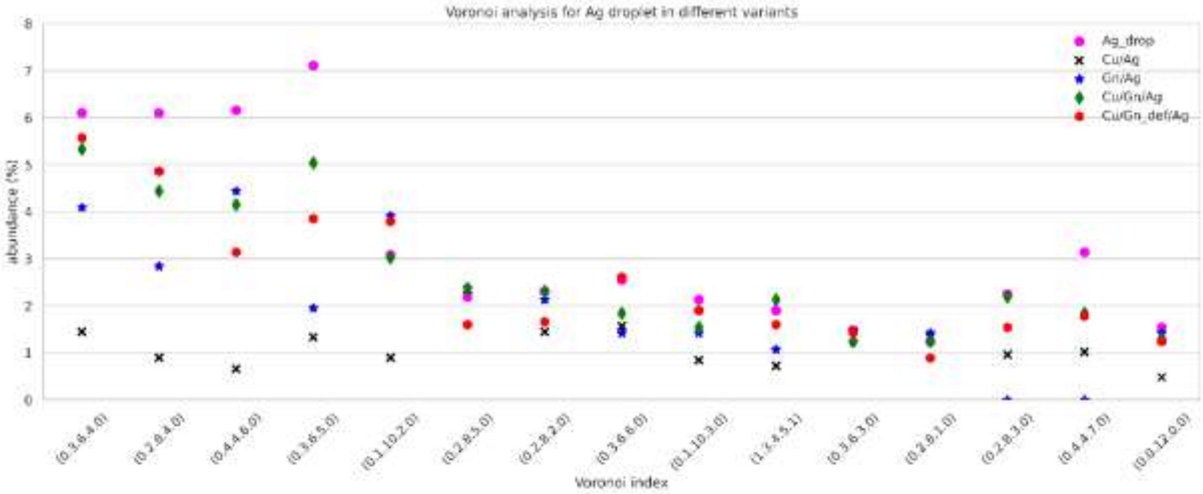

Figure S5 Comparison of VC types abundancy between three substrate variants and liquid Ag droplet.
